# Airway management: induced tension pneumoperitoneum

**DOI:** 10.11604/pamj.2016.25.125.9038

**Published:** 2016-10-31

**Authors:** Khedher Ahmed, El Ghali Mohamed Amine, Azouzi Abdelbaki, Ayachi Jihene, Meddeb Khaoula, Hamdaoui Yamina, Boussarsar Mohamed

**Affiliations:** 1 Medical Intensive Care Unit, Farhat Hached University Hospital, Sousse, Tunisia; 2 General Surgery Department, Farhat Hached University Hospital, Sousse, Tunisia

**Keywords:** Non-surgical pneumoperitoneum, barotraumas, acute abdominal compartment syndrome

## Abstract

Pneumoperitoneum is not always associated with hollow viscus perforation. Such condition is called non-surgical or spontaneous pneumoperitoneum. Intrathoracic causes remain the most frequently reported mechanism inducing this potentially life threatening complication. This clinical condition is associated with therapeutic dilemma. We report a case of a massive isolated pneumoperitoneum causing acute abdominal hypertension syndrome, in a 75 year female, which occurred after difficult airway management and mechanical ventilation. Emergent laparotomy yielded to full recovery. The recognition of such cases for whom surgical management can be avoided is primordial to avoid unnecessary laparotomy and its associated morbidity particularly in the critically ill.

## Introduction

Pneumoperitoneum (PP) commonly indicates a perforated hollow viscus that requires prompt surgical exploration and intervention [1]. However, cases of nonsurgical PP have also been reported. Ventilator induced barotrauma appears to be the most common underlying condition resulting in this kind of PP also termed spontaneous [1]. In these cases, the PP is often well tolerated and accompanied by pneumothorax and/or pneumomediastinum and/or subcutaneous emphysema. We report a case of isolated, compressive and poorly tolerated PP due to mechanical ventilation. This condition has opposed a double challenge, not only mechanism understanding but also management.

## Patient and observation

**Figure 1:**
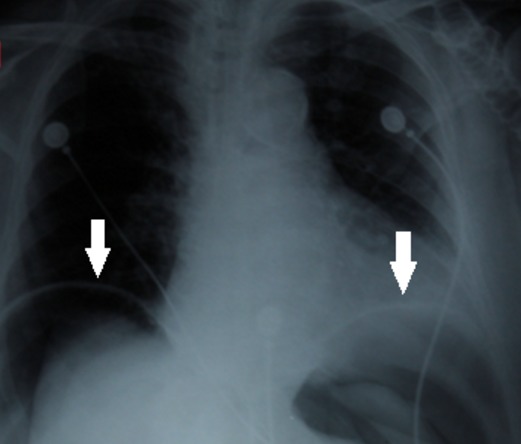
Chest X-ray at ICU admission after airway management and mechanical ventilation. It showed bilateral air collections in subphrenic areas (see arrows)

**Figure 2:**
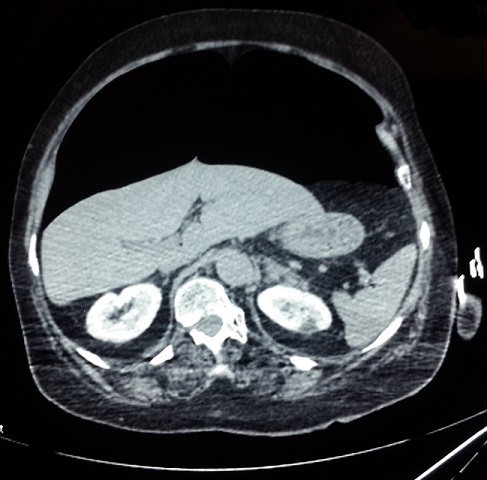
Thoraco-abdominal computed tomography scan performed one hour after the occurrence of the abdominal distension. It revealed a massive pneumoperitoneum, without intraperitoneal effusion, pneumothorax or pneumomediastinum

**Figure 3:**
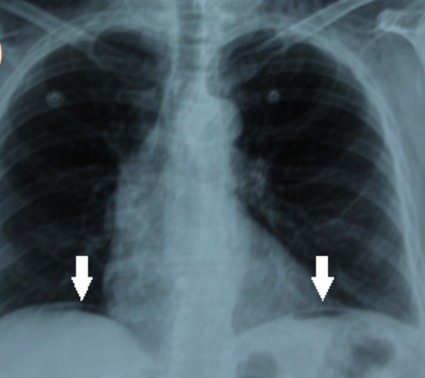
Chest X-ray at day 1 of surgical intervention. It showed significant regression of pneumoperitoneum (see arrows)

A 75-year-old female was admitted in the medical ICU for a sudden onset severe coma related to acute ischemic stroke complicating persistent atrial fibrillation after a short course in the cardiology department. She was obese (BMI, 36kg/m^2^). She had no surgical past history and she experienced no other recent complaints especially gastrointestinal. On first examination by the resuscitation team, the patient was in a comatose state with GCS at 3/15. She was afebrile. She had blood pressure at 90/40mmHg and heart rate at 140bpm. She had no dyspnea. White blood cells count was 7500/mm^3^ and CRP, 8mg/L. Airway management has been performed by a young trainee after a rapid sequence induction using hypnomidate (20mg) and suxamethonium (100mg). Mallampati classification [2] was graded III and Cormack-Lehane classification [3] graded III. At the first attempt the tube was misplaced in the esophagus, and then relayed as soon by endotracheal intubation. Mechanical ventilation under sedation was undertaken with assist-control ventilation (ACV) mode with a tidal volume set at 8ml/kg ideal body weight, respiratory rate at 16/min, FiO2 at 1 and PEEP (Positive End Expiratory Pressure) at 6cmH2O. After stabilization of the clinical condition the patient was transferred to the ICU. There was no per-procedural reported cough effort or valsalva–like maneuver. On immediate examination, the chest breath sounds were symmetrically transmitted. Peak airway inspiratory pressure was measured at 20cmH2O. Arterial blood gas analysis revealed, pH, 7.53 ; PaCO2, 28mmHg ; PaO2, 221mmHg, HCO3-, 21mmol/L. Lactates, 1.5mmol/L. Several minutes further, examination revealed a rapidly progressive abdominal distension with tympanism. There was no subcutaneous emphysema. Peak airway inspiratory pressure rose up to 33cmH2O. The chest X-ray showed bilateral collections of air in the subphrenic areas without pneumothorax nor pneumomediastinum (Figure 1). Thoraco-abdominal computed tomography scan was performed immediately and revealed a massive PP, without intraperitoneal effusion, pneumothorax nor pneumomediastinum (Figure 2). The clinical status evolved towards an intra-abdominal hypertension syndrome with hemodynamic instability, oliguria and ventilator asynchrony. Given the rapid worsening status, exploratory laparotomy was performed despite the lack of formal arguments in favor of viscus perforation. A sound of burst occurred when opening the peritoneum and a large quantity of pressurized air leaked out. There was no evidence of viscus perforation. A drainage tube was left in the peritoneal cavity. The immediate postoperative course was marked by a significant improvement of cardiorespiratory parameters. There was also a significant regression of PP at physical examination and persistence of a simple air crescent on the chest X-ray at day 1 (Figure 3) and complete resolution at day 3 of the postoperative period without recurrence. The patient died one month later in the ICU of a ventilator acquired pneumonia.

## Discussion

Non-surgical pneumoperitoneum (NSP) is a commonly described entity but not always recognized by physicians. Its prevalence is estimated at 5 to 15% [4]. Most cases of NSP occur as a procedural complication such as endoscopic procedures, peritoneal dialysis catheter placement or as a complication of medical intervention such as thoracic causes including ventilatory support and cardiopulmonary resuscitation [4]. Apart from the existence of diaphragmatic defects, the most likely mechanism of air entry into the peritoneum results from ruptured alveoli adjacent to the mediastinum. With the increasing pressure, the air dissects along anatomical fascial planes in the mediastinal structures into the retroperitoneum. The pressurized air then enters the peritoneal cavity through the mesentery [5]. This mechanism is called the “Macklin” effect [6]. In these cases, PP is usually well tolerated and associated with pneumothorax or pneumomediastinum [7]. In addition, it is closely correlated with underlying lung disease and ventilatory parameters settings, especially in ARDS (Acute respiratory Distress Syndrome) patients [8]. Another possible mechanism of PP results from tracheal rupture. This could be the consequence of thoracic trauma or iatrogenic lesions consecutive to tracheal or esophageal procedures [4]. The air dissects along the mediastinum to reach the peritoneum via diaphragmatic hiatus such as Larrey, Morgagni or Bochdalek hiatus.

The diagnostic approach of NSP is usually facilitated by the chronological relationship between the procedure likely to cause and its occurrence. Indeed, in the present case, the PP onset in the hour following the airway management and mechanical ventilation startup was very suggestive [5]. However, the fact that it was the only manifestation of barotrauma is rare, which represents one of the originalities of the reported case. The fact that, in the present case, there was neither per-procedural cough effort nor high airway pressure, prompted us to think that the origin of the air leak would have been a traumatic tracheal breach induced by the tracheal tube due to difficult airway management. This hypothesis was further enhanced as associated pneumothorax and pneumomediastinum were lacking.

Although laparotomy is warranted in the case of surgical PP, both as diagnostic and therapeutic procedure, it remains questionable in the case of NSP. Mularski conducted a literature review which included 196 cases of NSP, only 45 of them underwent laparotomy without evidence of perforated viscus [4]. The absence of peritoneal irritation and sepsis signs associated with minimal abdominal pain and distension was proposed as a suggestive picture of NSP for indicating conservative treatment [4–5]. In the present case, all these clinical and laboratory data conditions were present. Nevertheless, the rapid hemodynamic and respiratory destabilization caused by acute abdominal compartment syndrome was the most important determinant in decision-making to perform laparotomy. Indeed, rapid improvement occurred after the surgical evacuation of gas. A few similar cases have been reported in the literature [9]. Furthermore, we could a posteriori suggest that conservative treatment including advancing the tracheal tube near the carina, could have plugged the tracheal breach and therefore would have stopped the air supply to the peritoneal cavity. Then, percutaneous peritoneal cavity drainage could have been tried before going to the operating room [10]. The recognition of such cases in whom laparotomy can be avoided is important to prevent unnecessary surgery and its associated morbidity which could have ominous consequences mainly for critically ill patients.

## Conclusion

Non-surgical pneumoperitoneum is an uncommon entity. Intrathoracic causes especially during ventilatory support are the most frequently reported. Such cases are difficult to manage and create a major surgical dilemma. Surgeons and intensivists should be aware of the possible causes of NSP and should play the decisive role in preventing needless laparotomy in these patients. In cases of massive or tension PP and worsening respiratory and/or hemodynamic condition, percutaneous peritoneal cavity drainage could improve cardiopulmonary parameters and should be tried as a first line treatment.
